# *Porphyromonas gingivalis* outer membrane vesicles promote alveolar bone resorption by increasing the local RANKL/OPG ratio in experimental periodontitis model rats

**DOI:** 10.1038/s41598-026-46625-4

**Published:** 2026-04-05

**Authors:** Daisuke Nakamura, Yuji Inagaki, Yuta Uemura, Yuka Hiroshima, Yoshimune Iwata, Rie Kido, Mika Bando, Tomoko Sumitomo, Hiromichi Yumoto

**Affiliations:** 1 Department of Periodontology and Endodontology, Tokushima University Graduate School of Biomedical Sciences, 3-18-15 Kuramoto-cho, Tokushima, 770- 8504 Japan; 2 Department of Oral Microbiology, Tokushima University Graduate School of Biomedical Sciences, 3-18-15 Kuramoto-cho, Tokushima, 770-8504 Japan

**Keywords:** *Porphyromonas gingivalis*, Outer membrane vesicles, Periodontitis, Bone resorption, Osteoclastogenesis, RANKL/OPG, Cell biology, Diseases, Medical research, Microbiology

## Abstract

Outer membrane vesicles (OMVs) released by bacteria contain bacteria-specific nucleic acids and proteins. *Porphyromonas gingivalis* (*Pg*)-OMVs exert various effects on periodontal tissue cells; however, their involvement in bone metabolism remains unclear. Therefore, this study aimed to investigate the effects of *Pg*-OMVs on osteoclasts and osteoblasts in vitro and elucidate their involvement in alveolar bone resorption in vivo using experimental periodontitis model rats. Tartrate-resistant acid phosphatase staining and Western blotting revealed that *Pg*-OMVs promoted osteoclast differentiation and increased the levels of several osteoclast differentiation markers, respectively, in RAW264.7 cells. Quantitative reverse transcription-polymerase chain reaction and Western blotting revealed that *Pg*-OMVs increased the receptor activator of nuclear factor-kappa B (NF-κB) ligand (RANKL) levels and decreased osteoprotegerin (OPG) levels in MC3T3-E1 cells. *Pg*-OMVs reduced alkaline phosphatase activity and calcified nodule formation in a concentration-dependent manner. Rats were divided into four groups (healthy control, *Pg*-OMV administration, ligature-induced periodontitis, and ligature-induced periodontitis with *Pg*-OMV administration groups), and the alveolar bone resorption degree was measured via micro-computed tomography. *Pg*-OMVs administration significantly promoted alveolar bone resorption and increased the RANKL/OPG ratio in periodontal tissues. Our results suggest that *Pg*-OMVs contribute to alveolar bone resorption by promoting osteoclast differentiation, suppressing osteoblast mineralization, and increasing the RANKL/OPG ratio.

**Supplementary Information:**

The online version contains supplementary material available at 10.1038/s41598-026-46625-4.

## Introduction

Periodontitis, a chronic inflammatory disease, is primarily initiated by periodontal pathogenic bacteria in dental plaque and exacerbated by various factors, such as occlusal trauma and systemic conditions^1^.

Increased abundance of periodontal pathogens, including *Porphyromonas gingivalis* (*Pg*), *Tannerella forsythia*, *Treponema denticola*, *Aggregatibacter actinomycetemcomitans*, *Prevotella intermedia*, and *Fusobacterium nucleatum* (*Fn*), triggers inflammatory responses in periodontal tissues by activating the innate immune system and promoting pro-inflammatory cytokine production^2^. Persistent challenges with these bacteria lead to progressive bone and soft tissue destruction, ultimately resulting in tooth loss. Periodontitis stimulates the immune system in both periodontal lesions and systemic tissues^3^. Chronic periodontitis is strongly associated with various systemic inflammatory diseases and conditions, such as diabetes mellitus and rheumatoid arthritis, and cardiovascular diseases, such as coronary heart disease and stroke^4–8^.

*Pg* plays a pivotal role in the development of periodontitis and is considered a key pathogen among disease-provoking periodontal microbiota, as it remodels the commensal microbiota into a dysbiotic state by disrupting host homeostasis^9^. *Pg* expresses several virulence factors, including lipopolysaccharides (LPSs), capsular polysaccharides, fimbriae, and gingipains, which are cell surface cysteine proteinases^10^. Among these factors, *Pg*-LPS is a potent stimulator of pro-inflammatory responses and bone resorption, significantly exacerbating periodontitis by promoting the production of pro-inflammatory cytokines, such as interleukin (IL)−1α, IL-1β, IL-6, IL-8, IL-18, and tumor necrosis factor (TNF)-α^10,11^.

Most gram-negative bacteria, including oral species *Pg*, *A. actinomycetemcomitans*, and *Fn*, produce outer membrane vesicles (OMVs), which are surface-bound or cell-free spherical structures formed at various growth stages under different environmental conditions^12,13^. OMVs are approximately 20–250 nm in size and contain several bacterial components, including LPSs, periplasmic and membrane-bound proteins, enzymes, toxins, DNA, RNA, and peptidoglycan^12,14^. They are involved in nutrient acquisition, stress adaptation, and communication with other bacterial and host cells^15–17^. They play roles similar to those of the parent bacterium, with many of their components contributing to the mediation of adherence, host cell invasion and destruction, modulation and dysregulation of host immune responses, and biofilm formation^12^. However, compared to the parent bacterium, OMVs have an advantage in that their enclosed components are protected from proteolytic degradation and can be delivered over long distances to tissues and organs^17,18^. Consequently, OMVs are suggested to play significant roles in the pathogeneses of systemic diseases associated with the parent bacterium^15,19^.

*Pg*-OMVs exhibit several pathogenic mechanisms. They exert adhesive effects by promoting bacterial adhesion between non-co-aggregating species, invasive effects by interacting with the surface receptors on host cells, and immunomodulatory effects, leading to host cell damage, dysfunction, and even cell death^20^. Proteomic analyses have revealed that *Pg*-OMVs contain various virulence factors, including fimbriae protein A, peptidylarginine deiminase, hemagglutinin A, gingipains, and heat shock proteins, which are implicated in the pathogenesis of periodontitis^20,21^. Notably, several of these virulence factors are present at higher levels in *Pg*-OMVs than in *Pg* bacterial cells. For example, gingipain levels are approximately 3–5-times higher in *Pg*-OMVs than in the parent bacterial cells^22^.

*Pg*-OMVs enter the bloodstream and negatively affect distant tissues and organs via systemic circulation^23^. Many studies have reported a correlation between *Pg*-OMVs and *Pg* infection-associated systemic diseases^24–28^. For example, gingipain-containing *Pg*-OMVs accumulate in the liver, where they inhibit insulin-stimulated glycogen synthesis and contribute to diabetes mellitus progression^29^.

Alveolar bone loss, which is induced by host immune and inflammatory responses to microbial challenges, is a hallmark of periodontitis progression^30–32^. Osteoclasts, multinucleated cells (MNCs) derived from the monocyte/macrophage lineage, are the principal cells responsible for bone resorption. Pathological bone resorption is driven by the stimulation of osteoclast differentiation and formation and regulated by several transcription factors and enzymes, including nuclear factor of activated T-cells 1 (NFATc1), dendritic cell-specific transmembrane protein (DC-STAMP), tartrate-resistant acid phosphatase (TRAP), and Cathepsin K^33–37^. Osteoblasts regulate osteoclastogenesis by producing receptor activator of nuclear factor-kappa B (NF-κB) ligand (RANKL), an essential cytokine interacting with its receptor RANK on osteoclast precursors^38^. Osteoprotegerin (OPG), a decoy receptor for RANKL, is predominantly produced by osteoblasts and inhibits osteoclastogenesis by preventing RANKL–RANK interaction^39^. In inflamed periodontal tissues, RANKL/OPG ratio increases due to elevated RANKL levels, reduced OPG levels, or both. This increased RANKL/OPG ratio possibly drives osteoclastogenesis^38^. Chen et al. reported that *Fn*-OMVs increase the number of osteoclasts and promote alveolar bone loss in periodontitis model mice^40^. However, pathogenic effects of *Pg*-OMVs on RANKL–RANK interactions and the RANKL/OPG ratio during the onset and progression of periodontitis remain unknown.

Increasing evidence suggests that *Pg*-OMVs play an important role in the pathogenesis of periodontitis^15,17,19^. We previously demonstrated that *Pg*-OMVs activate the extracellular signal-regulated protein kinase-1/2, c-Jun N-terminal kinase, p38 mitogen-activated protein kinase, stimulator of interferon gene, and NF-κB signaling pathways, leading to increased IL-6 and IL-8 expression levels in human gingival epithelial cells^41^. This finding suggests that *Pg*-OMVs contribute to the exacerbation of periodontitis by stimulating multiple signaling pathways. Although *Pg*-OMVs exert various effects on periodontal tissue component cells, their specific roles in alveolar bone resorption remains unclear. In this study, we investigated the effects of *Pg*-OMVs on murine preosteoblastic and osteoclast precursor cells in vitro and evaluated their involvement in alveolar bone resorption in vivo using experimental periodontitis model rats.

## Results

### Effect of *Pg*-OMVs on RAW264.7 and MC3T3-E1 cell proliferation

**Fig. 1:**
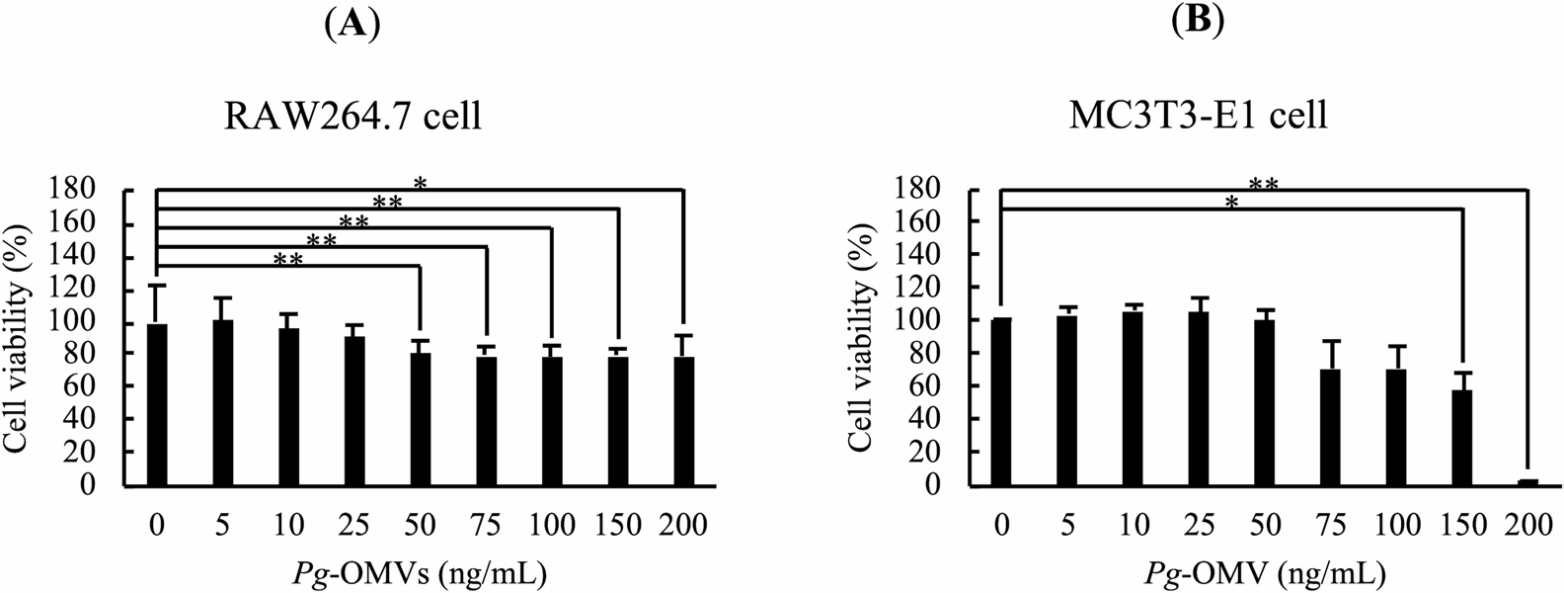
Effect of *Pg*-OMVs on the cell viability of RAW264.7 and MC3T3E-1. RAW264.7 and MC3T3-E1 cells were seeded at a density of 5,000 cells/well in 96-well plates. **A** RAW264.7 cells were cultured in the presence of sRANKL and treated with the indicated concentrations of *Pg*-OMVs for 48 h. **B** MC3T3-E1 cells were treated with *Pg*-OMVs for 48 h. Cell viability was assessed using Cell Counting Kit-8. Data are expressed as percentages relative to the respective controls. Data are presented as mean ± SD from three independent biological replicates (*n* = 3). *P < 0.05 and **P < 0.01 vs. the sRANKL-stimulated control (50 ng/mL sRANKL, 0 ng/mL *Pg*-OMVs) for RAW264.7 cells; *P < 0.05 and **P < 0.01 vs. the untreated control (0 ng/mL *Pg*-OMVs) for MC3T3-E1 cells.

The inhibitory effects of *Pg*-OMVs on RAW264.7 and MC3T3-E1 cell proliferation were investigated after 48 h of culture. Notably, in the presence of sRANKL, RAW264.7 cell viability was significantly decreased by *Pg*-OMVs at concentrations ≥ 50 ng/mL compared with the control (0 ng/mL *Pg*-OMVs) (Fig. 1A). In contrast, *Pg*-OMVs at concentrations ≥ 75 ng/mL decreased MC3T3-E1 cell viability, with significant differences observed at 150 and 200 ng/mL compared with the control (Fig. 1B).

### *Pg*-OMVs promote osteoclast differentiation and increase osteoclast marker levels in RAW264.7 cells

To investigate the effect of *Pg*-OMVs on osteoclast differentiation, RAW264.7 cells were cultured in the presence or absence of sRANKL and treated with *Pg*-OMVs (0, 10, and 100 ng/mL) for 5 days.

**Fig. 2:**
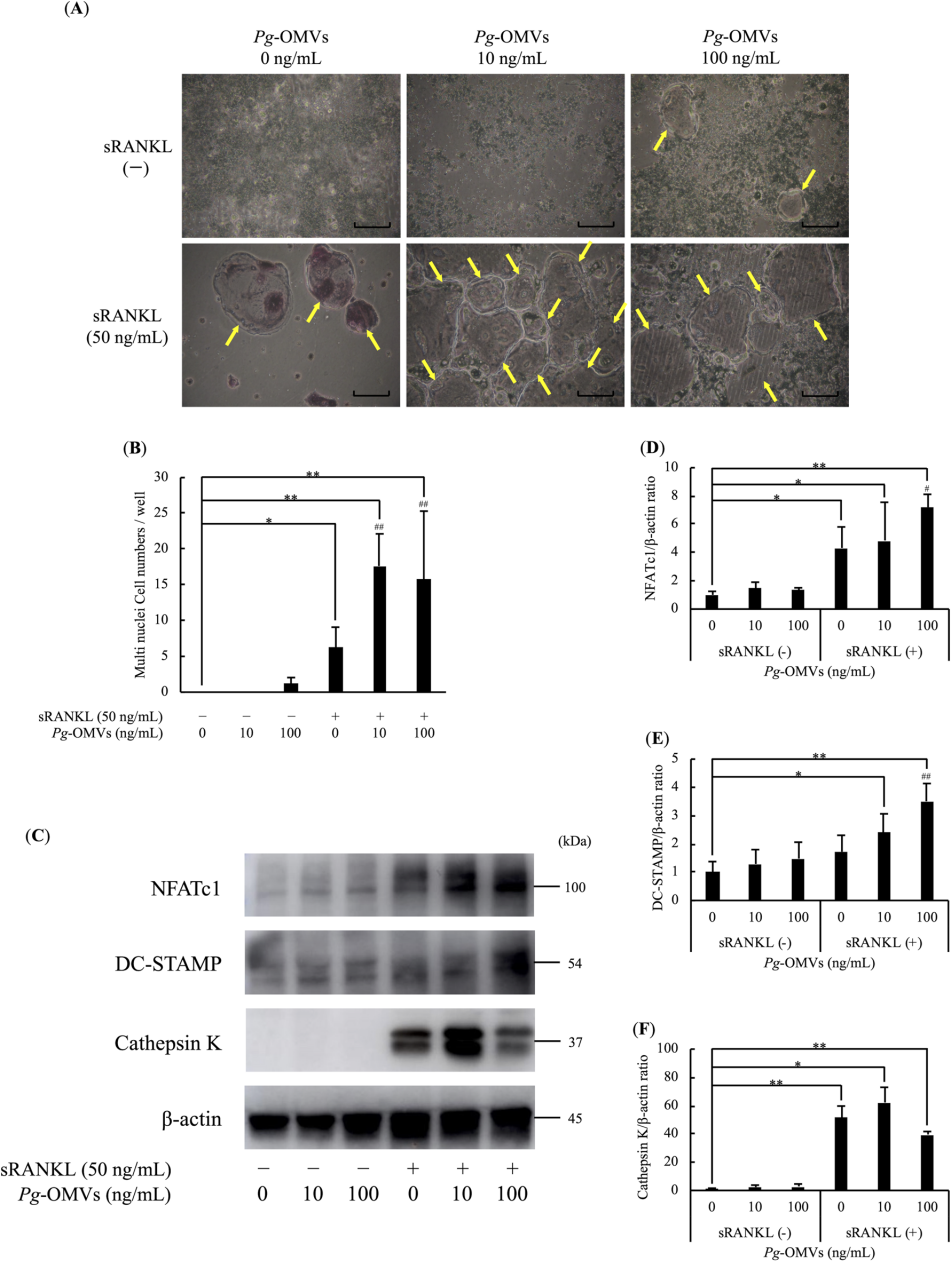
Effects of *Pg*-OMVs on osteoclast differentiation and osteoclast-specific marker expression in RAW264.7 cells. **A** Representative microscopic TRAP staining images of RAW264.7 cells cultured for 5 days in the presence or absence of sRANKL (50 ng/mL) and treated with *Pg*-OMVs (0, 10, or 100 ng/mL). Upper panels: sRANKL (−); lower panels: sRANKL (+). Scale bars: 300 μm. **B** Quantitative analysis of TRAP-positive MNCs with ≥ 3 nuclei per well under each condition. **C–E** Expression levels of NFATc1, DC-STAMP, and Cathepsin K in RAW264.7 cells cultured under the same conditions as above for 5 days, measured via Western blotting. β-actin served as a loading control. All bands were derived from the same gel; membranes were cut horizontally after transfer or stripped and reprobed with the indicated antibodies. Data are presented as mean ± SD from three independent biological replicates (*n* = 3). *P < 0.05 and **P < 0.01 vs. the sRANKL-unstimulated control (0 ng/mL sRANKL, 0 ng/mL *Pg*-OMVs); ^#^*P* < 0.05 and ^##^*P* < 0.01 vs. the sRANKL-stimulated control (50 ng/mL sRANKL, 0 ng/mL *Pg*-OMVs).

Upon stimulation with sRANKL, RAW264.7 cells differentiated into TRAP-positive MNCs and osteoclasts. *Pg*-OMVs (10 ng/mL) increased the number and size of osteoclasts under sRANKL-stimulated conditions; however, osteoclast numbers tended to decrease at 100 ng/mL *Pg*-OMVs, although the difference was not statistically significant (Fig. 2A and B). Even without sRANKL stimulation, 100 ng/mL *Pg*-OMVs showed a slight increase in osteoclast differentiation; however, the difference was not statistically significant compared with the unstimulated control (0 ng/mL sRANKL, 0 ng/mL *Pg*-OMVs) (Fig. 2B). These results indicate that *Pg*-OMVs enhance sRANKL-induced osteoclast differentiation in vitro.

To further elucidate the molecular mechanisms underlying osteoclastogenesis promotion by *Pg*-OMVs, we examined their effects on the expression levels of osteoclast differentiation markers via Western blotting analysis. In RAW264.7 cells without sRANKL stimulation, *Pg*-OMVs did not significantly increase the protein levels of osteoclast differentiation markers, including NFATc1, DC-STAMP, and Cathepsin K, compared with the unstimulated control (Fig. 2C–F). In sRANKL-induced osteoclasts, *Pg*-OMVs (100 ng/mL) significantly increased the expression of NFATc1 and DC-STAMP compared with the sRANKL-stimulated control (50 ng/mL sRANKL, 0 ng/mL *Pg*-OMVs) (Fig. 2C–E). Furthermore, *Pg*-OMVs at 10 ng/mL tended to increase the expression of Cathepsin K compared with the sRANKL-stimulated control, whereas a high concentration of *Pg*-OMVs (100 ng/mL) result in expression levels comparable to the control (Fig. 2C and F).

### *Pg*-OMVs increase the RANKL/OPG ratio in MC3T3-E1 cells

**Fig. 3:**
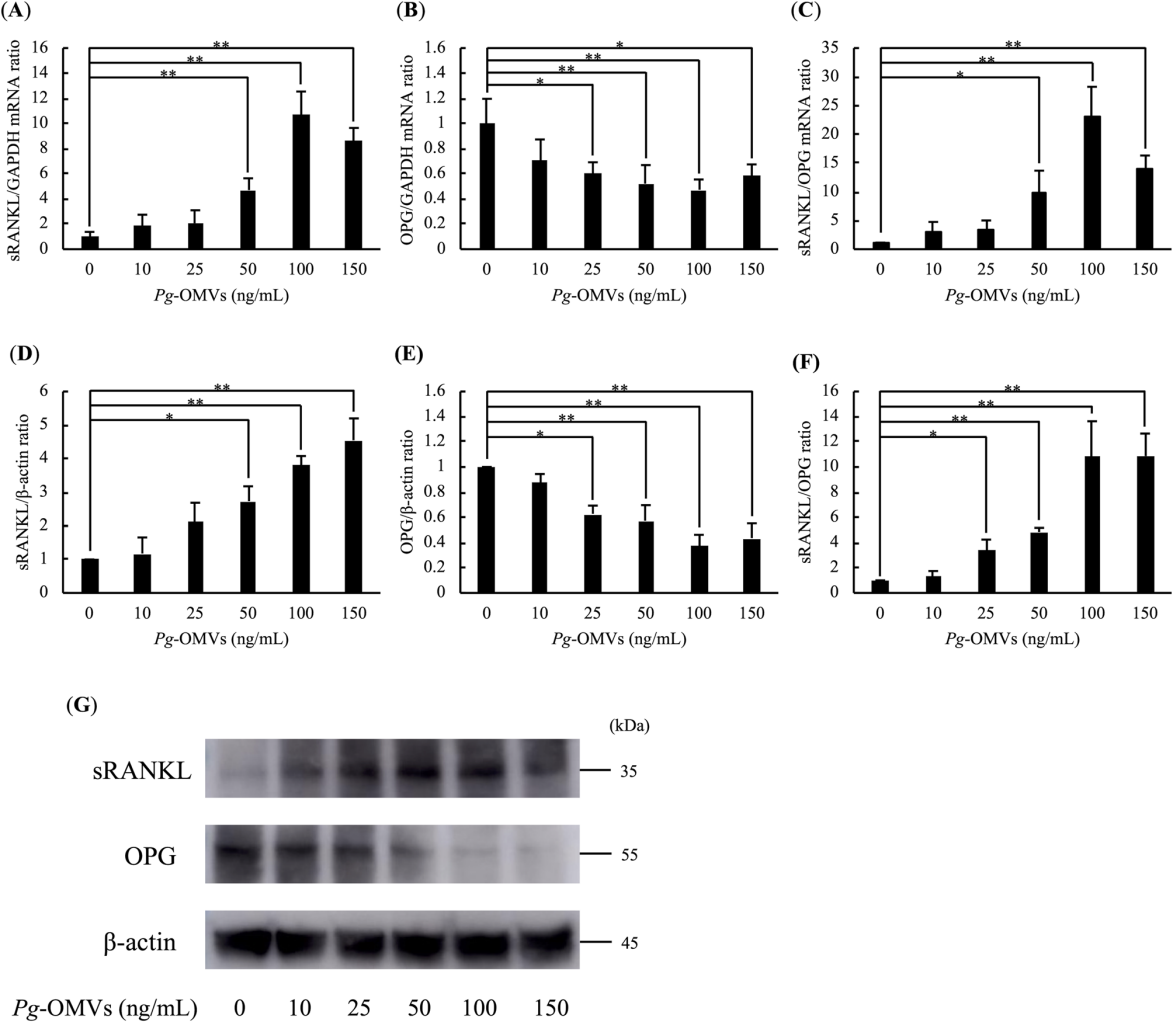
Effects of *Pg*-OMVs on RANKL and OPG expression in MC3T3-E1 cells. **A–C** Relative mRNA levels of RANKL and OPG were determined via qRT-PCR. **D–G** Protein levels of RANKL and OPG were measured via Western blotting. β-actin served as a loading control. Membranes were stripped and reprobed with the indicated antibodies. All bands shown were derived from the same gel. Data are presented as mean ± SD from three independent biological replicates (*n* = 3). *P < 0.05 and **P < 0.01 vs. untreated control (0 ng/mL *Pg*-OMVs).

Given the role of osteoblast-derived RANKL and OPG in osteoclast differentiation, we investigated the effects of *Pg*-OMVs on the RANKL/OPG balance in MC3T3-E1 cells. MC3T3-E1 cells were cultured in calcification-inducing medium and treated with *Pg*-OMVs (10–150 ng/mL) for 4 days. Because MC3T3-E1 cells were used to evaluate regulation of endogenous RANKL and OPG expression, exogenous RANKL stimulation was not applied. Cells were treated with a range of *Pg*-OMVs concentrations (10–150 ng/mL), including levels associated with reduced cell viability (Fig. 1B), to assess dose-dependent responses. *Pg*-OMVs significantly increased *Tnfsf11* (RANKL) mRNA expression at concentrations ≥ 50 ng/mL and decreased *Tnfrsf11b* (OPG) mRNA expression at concentrations ≥ 25 ng/mL (Fig. 3A, B). In agreement with the mRNA expression, Western blotting analysis showed that *Pg*-OMVs increased RANKL protein expression and decreased OPG protein expression (Fig. 3G), which was confirmed by densitometric analysis (Fig. 3D and E). *Pg*-OMVs at concentrations ≥ 50 ng/mL significantly increased RANKL protein expression, whereas concentrations ≥ 25 ng/mL significantly decreased OPG protein expression (Fig. 3D and E). Consequently, *Pg*-OMVs increased the RANKL/OPG ratio in MC3T3-E1 cells (Fig. 3C, F).

### *Pg*-OMVs inhibit alkaline phosphatase (ALP) activity and extracellular matrix mineralization in MC3T3-E1 cells

**Fig. 4:**
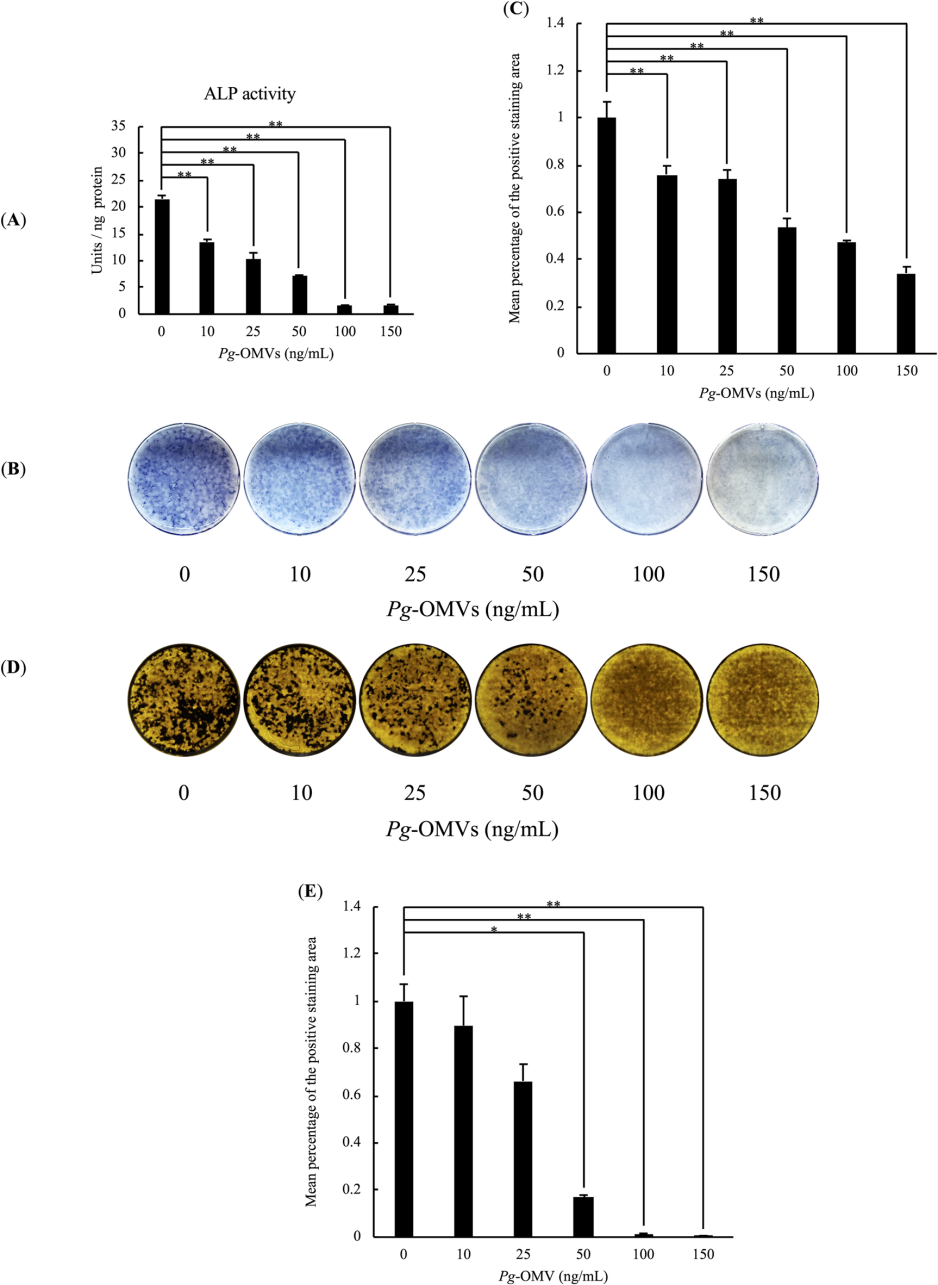
Effects of *P*g-OMVs on ALP activity and calcified nodule formation in MC3T3-E1 cells. **A** ALP activity was quantified using an ALP activity assay kit. **B** Representative images of ALP staining in *Pg*-OMVs-treated MC3T3-E1 cells. **C** Quantification of ALP-stained area using ImageJ. **D** Representative images of von Kossa staining in *Pg*-OMVs-treated cells. **E** Quantification of calcified nodule area using ImageJ. Data are presented as mean ± SD from three independent biological replicates (*n* = 3). *P < 0.05 and **P < 0.01 vs. untreated control (0 ng/mL *Pg*-OMVs).

To investigate the effects of *Pg*-OMVs on osteoblast differentiation and extracellular matrix mineralization, MC3T3-E1 cells were cultured with various concentrations of *Pg*-OMVs in a calcification-inducing medium. *Pg*-OMVs significantly attenuated ALP enzymatic activity in cell lysates in a concentration-dependent manner (Fig. 4A). Consistently, ALP staining of culture plates demonstrated a marked suppression of ALP activity by *Pg*-OMVs (Fig. 4B and C). Furthermore, *Pg*-OMVs significantly inhibited mineralized nodule formation in MC3T3-E1 cells, as assessed by mineralization staining (Fig. 4D and E).

### Effects of *Pg*-OMVs on osteoclast differentiation in a RAW264.7–MC3T3-E1 co-culture system

**Fig. 5:**
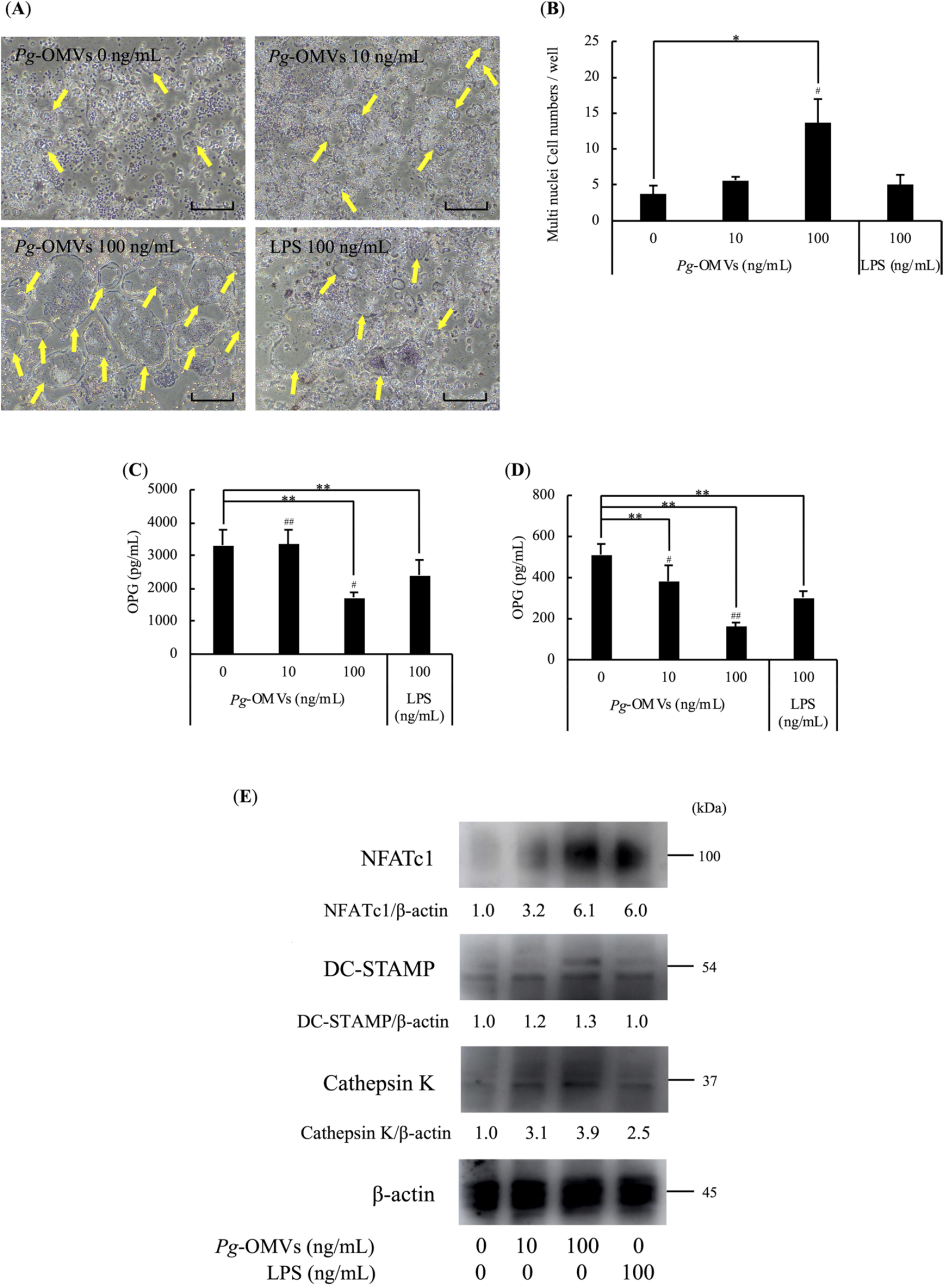
Effects of *Pg*-OMVs on osteoclast differentiation via MC3T3-E1 cell–derived OPG in a RAW264.7–MC3T3-E1 co-culture system. **A** Representative microscopic images of TRAP-stained RAW264.7 cells showing TRAP-positive MNCs. Scale bar = 300 μm. **B** Quantitative analysis of TRAP-positive MNCs containing three or more nuclei per well. **C** and **D** On day 5 of co-culture, OPG concentrations in culture supernatants collected separately from the upper (C) and lower (D) chambers were measured by ELISA. Data are presented as mean ± SD from three independent biological replicates (*n* = 3). *P < 0.05 and **P < 0.01 vs. untreated control (0 ng/mL *Pg*-OMVs); #*P* < 0.05 and ##*P* < 0.01 vs. *Pg*-LPS (100 ng/mL). (**E**) Expression of osteoclast differentiation markers was assessed by Western blotting. Representative images showing NFATc1, DC-STAMP, and Cathepsin K. Similar results were obtained in two independent experiments. β-actin was used as a loading control. All bands were derived from the same gel; membranes were cut horizontally after transfer or stripped and reprobed with the indicated antibodies.

We examined whether *Pg*-OMVs modulate osteoclast differentiation via MC3T3-E1 cell–derived OPG using a RAW264.7–MC3T3-E1 co-culture system under sRANKL stimulation. RAW264.7 cells were co-cultured with MC3T3-E1 cells using a cell insert system and treated with *Pg*-OMVs or *Pg*-LPS. TRAP staining revealed that treatment with 100 ng/mL of *Pg*-OMVs markedly enhanced sRANKL-induced osteoclast differentiation, as evidenced by an increase in both the number and size of TRAP-positive MNCs, whereas 10 ng/mL of *Pg*-OMVs and 100 ng/mL of *Pg*-LPS did not significantly affect osteoclast formation (Fig. 5A and B).

To clarify whether these effects were associated with alterations in OPG production, OPG levels in culture supernatants were separately measured in the upper and lower chambers. ELISA analysis demonstrated that treatment with 100 ng/mL of *Pg*-OMVs significantly reduced OPG levels in both chambers compared with the untreated control and *Pg*-LPS–treated groups (Fig. 5C and D).

Consistent with the enhanced osteoclast formation, Western blot analysis showed that treatment with 100 ng/mL of *Pg*-OMVs increased the expression of osteoclast differentiation markers, including NFATc1, DC-STAMP, and Cathepsin K, in RAW264.7 cells compared with treatment with 100 ng/mL of *Pg*-LPS (Fig. 5E).

### *Pg*-OMVs induce and promote alveolar bone resorption in experimental periodontitis model rats

Next, to elucidate the roles of *Pg*-OMVs in bone resorption in periodontitis, the distance from the cementoenamel junction (CEJ) to alveolar bone crest (ABC) of the second molar was measured in all groups, and alveolar bone resorption in rat maxilla was analyzed via µCT. On µCT images, no significant differences in bone resorption around the left second molar (untreated side) were observed among the groups. In contrast, bone resorption around the right second molar (ligature side) was greater than that around the left second molar in the ligature control, OMV administration, and ligature with OMV administration groups. Furthermore, bone resorption around the right second molar was significantly higher in these three experimental groups than in the healthy control group (Fig. 5A, B). To examine whether *Pg*-OMVs induce bone resorption in periodontitis, alveolar bone erosion was compared between the OMV administration and healthy control groups. The distance between CEJ and ABC was 273.0 ± 38.8 μm in the healthy control group and 511.2 ± 126.8 μm in the OMV administration group (Fig. 5B). *Pg*-OMVs administration significantly increased bone resorption by approximately 87.3% compared to that in the untreated healthy control group.

To further examine whether *Pg*-OMVs accelerate bone resorption in periodontitis, alveolar bone erosion was compared between the ligature with OMV administration and ligature control groups. The distance was 900.0 ± 46.7 μm in the ligature with OMV administration group and 699.7 ± 60.9 μm in the ligature control group (Fig. 5B). Therefore, *Pg*-OMVs administration significantly increased bone resorption by approximately 28.6% compared to ligature-induced periodontitis alone (*p* < 0.05). Furthermore, Bone Volume/Total (Tissue) Volume (BV/TV) analysis confirmed significant alveolar bone resorption in all three experimental groups compared with the control group (*p* < 0.01) (Fig. 5C and D). The ligature with OMV administration group exhibited the lowest BV/TV values (44.3 ± 6.3%), consistent with the increased CEJ–ABC distance relative to the OMV administration (56.1 ± 4.8%) and ligature control groups (53.2 ± 4.1%) (*p* < 0.01 and *p* < 0.05, respectively).

### *Pg*-OMVs increase the RANKL/OPG ratio in the periodontal tissues of experimental periodontitis model rats

**Fig. 6:**
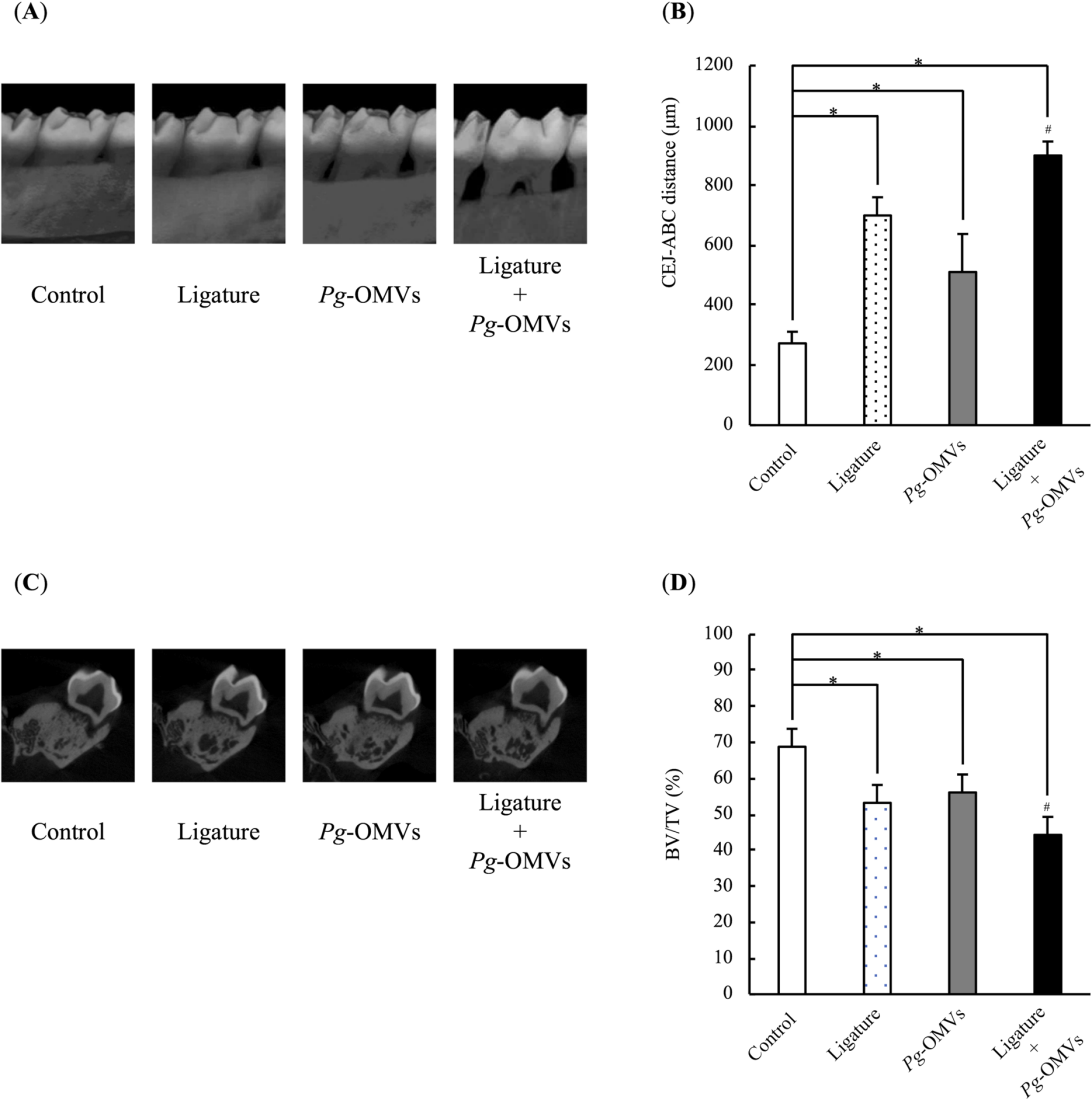
Effect of *Pg*-OMVs on bone resorption in ligature-induced periodontitis. (**A**) Representative 3D reconstruction images of the sagittal views of the maxillary second molars in rats obtained via µCT. (**B**) Distance from the CEJ to the ABC, used as a marker of alveolar bone resorption, determined via µCT. (**C**) Representative µCT images of the coronal views of the furcation area of the maxillary second molars. (**D**) Bone volume fraction (BV/TV) of the furcation area on the buccal side of the second molar, analyzed using CTan software. Data are presented as mean ± SD (*n* = 5 per group). *P < 0.01 vs. Control (healthy control group); ^#^*P* < 0.01 vs. Ligature (ligature control group).

The RANK–RANKL–OPG system is a major regulatory pathway for osteoclast differentiation and activation in bone tissues. To assess the effects of *Pg*-OMVs on the RANKL/OPG balance in vivo, RANKL and OPG expression levels were examined in periodontal tissues. Western blot analysis of gingival tissue lysates revealed that RANKL protein levels were significantly increased, whereas OPG protein levels were significantly decreased, in the OMV administration and ligature with OMV administration groups (Fig. 6A–C). Consequently, local administration of *Pg*-OMVs resulted in an increased RANKL/OPG ratio in periodontal tissues (Fig. 6D). IL-6 and TNF-α protein levels in gingival tissues were significantly increased in all three experimental groups compared with the healthy control group, with the highest TNF-α level observed in the ligature with OMV administration group (Fig. 6E, F).

**Fig. 7:**
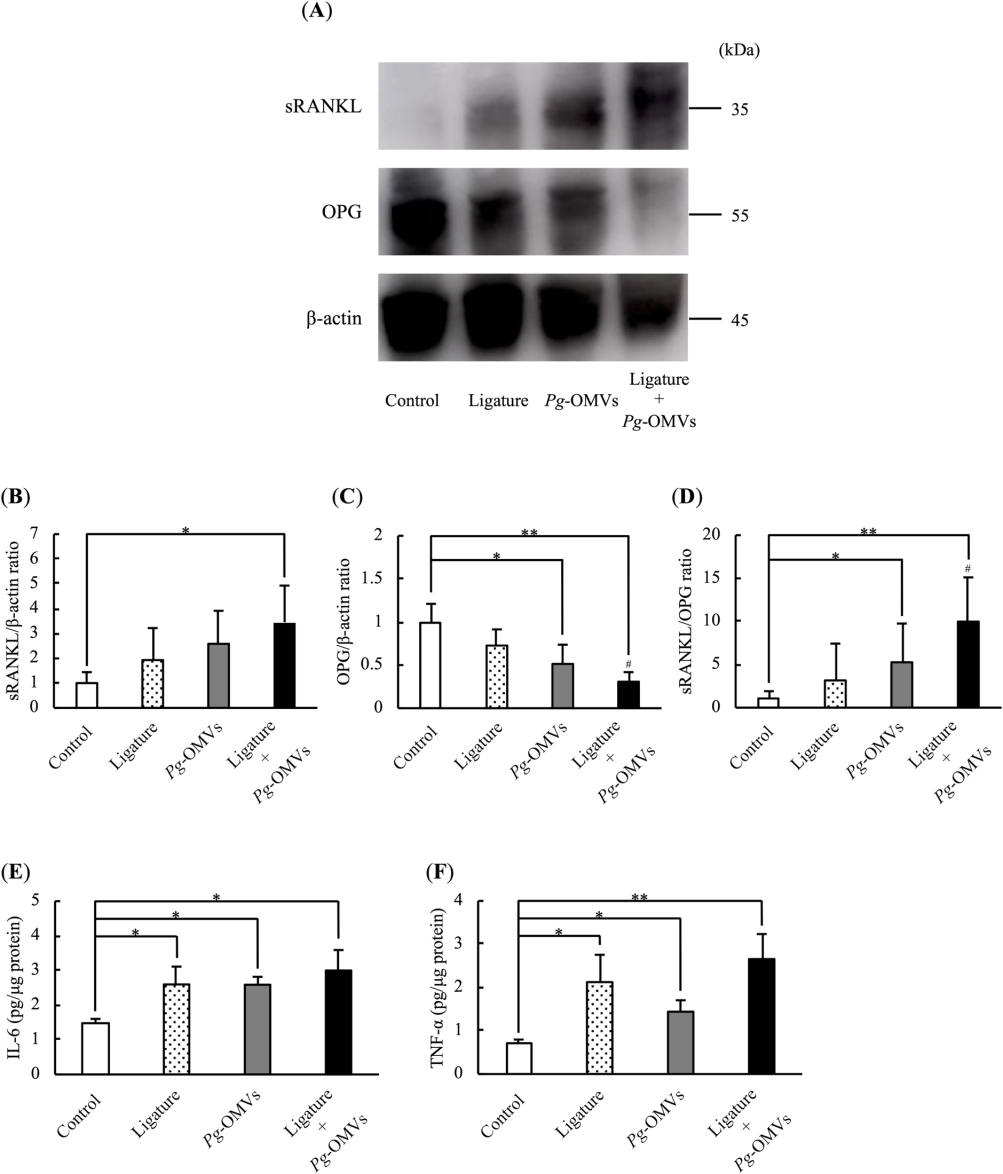
Effects of *Pg*-OMVs on RANKL, OPG, IL-6, and TNF-α expression levels in rat periodontal tissues. (**A–D**) Protein levels of RANKL, OPG, and β-actin in the tissues were measured by Western blotting. Membranes were stripped and reprobed with the indicated antibodies. All bands shown were derived from the same gel. (**E–F**) IL-6 and TNF-α levels in the tissues were measured via ELISA. Data are presented as mean ± SD (*n* = 5 per group). *P < 0.05 and **P < 0.01 vs. Control (healthy control group); ^#^*P* < 0.01 vs. Ligature (ligature control group).

Serum levels of NTx-1, a marker of bone resorption, were measured to assess the systemic effects of *Pg*-OMVs on osteoclast differentiation in vivo. Serum NTx-1 levels were significantly higher in the ligature with OMV administration group than in the other groups, whereas no significant differences were observed among the remaining three groups (Fig. 7).

## Discussion

In this study, we investigated the role of *Pg*-OMVs in alveolar bone resorption associated with periodontitis. To determine the role of *Pg*-OMVs in osteoclastogenesis, we examined the effects of *Pg*-OMVs on pre-osteoclastic cell differentiation using sRANKL-treated murine osteoclast precursor RAW264.7 cells. Both low and high concentrations of *Pg*-OMVs enhanced sRANKL-induced osteoclast differentiation in RAW264.7 cells. Notably, *Pg*-OMVs differentially modulated osteoclast differentiation markers in sRANKL-induced osteoclasts (Fig. 2), with robust induction of NFATc1 and DC-STAMP observed at high concentration compared with the sRANKL-stimulated control, whereas Cathepsin K expression showed only a modest increase at the low concentration and remained comparable to the sRANKL-stimulated control at the high concentration. These findings suggest that *Pg*-OMVs may differentially influence the early commitment and late maturation stages of osteoclast differentiation. Moreover, *Pg*-OMVs at concentrations ≥ 50 ng/mL gradually decreased RAW264.7 cell viability. Therefore, the relatively weak inductive effects of high concentrations of *Pg*-OMVs on sRANKL-induced osteoclast differentiation and Cathepsin K expression may be attributable, at least in part, to their inhibitory effects on cell proliferation.

When pre-osteoclastic cells are stimulated by RANKL–RANK interactions, NFATc1 translocates from the cytoplasm to the nucleus and functions as an active transcription factor^37^. Previous studies have demonstrated that NFATc1 plays a central role in osteoclast differentiation by regulating the expression of osteoclast-related genes, including *Acp5* (TRAP), *Dcstamp* (DC-STAMP), and *Ctsk* (Cathepsin K)^37,42,43^. Consistent with these findings, our results suggest that *Pg*-OMVs enhance NFATc1 expression under sRANKL stimulation, thereby promoting the transcription of osteoclast-related genes and osteoclast differentiation.

Osteoblasts regulate osteoclast differentiation by producing RANKL and its decoy receptor OPG. In the present study, *Pg*-OMVs increased RANKL expression and decreased OPG expression in MC3T3-E1 osteoblastic cells at both the mRNA and protein levels, resulting in a marked increase in the RANKL/OPG ratio. The RANK–RANKL–OPG signaling pathway plays a central role in bone resorption by tightly controlling osteoclast differentiation and activation^44^. While individual *Pg*-derived virulence factors, such as *Pg*-LPS and gingipains, have been reported to modulate RANKL and OPG expression in osteoblasts^45–47^, the effects of *Pg*-OMVs on osteoblast-mediated regulation of osteoclastogenesis have not been fully elucidated. In the present study, our findings indicate that *Pg*-OMVs, which deliver multiple virulence factors, indirectly promote osteoclastogenesis by altering the RANKL/OPG balance through modulation of osteoblast-derived signals. These results suggest that *Pg*-OMVs may function as a key mediator of osteoblast–osteoclast interaction and thereby contribute to alveolar bone destruction associated with periodontitis.

Moreover, *Pg*-OMVs reduced ALP activity and calcified nodule formation in MC3T3-E1 cells in a concentration-dependent manner (Fig. 4). As ALP activity is essential for osteoblast-mediated mineralization^48^, these findings indicate that *Pg*-OMVs impair the osteogenic capacity of osteoblasts. Such impairment may represent an additional mechanism by which *Pg*-OMVs contribute to impaired bone formation and alveolar bone destruction associated with periodontitis. Yan et al.. reported that high concentrations of *Pg*-OMVs (5 and 10 µg/mL) inhibit osteogenic differentiation via the serum amyloid A3–mediated Toll-like receptor 4/MyD88/NF-κB signaling pathway^49^. In contrast, our study demonstrated inhibitory effects on osteogenic activity at substantially lower *Pg*-OMVs concentrations, suggesting that distinct or additional signaling mechanisms may be involved. The precise molecular pathways underlying these effects remain to be elucidated and warrant further investigation. Under inflammatory bone destruction conditions, such as periodontitis, bacterial components including LPS are known to impair osteoblast function and contribute to enhanced bone resorption. In this study, our findings suggest that *Pg*-OMVs may modulate osteoblast function and osteoclast differentiation, thereby contributing to bone resorption in periodontitis.

In the present study, we demonstrated that *Pg*-OMVs modulate osteoclast differentiation in a non-contact RAW264.7–MC3T3-E1 co-culture system, accompanied by a decrease in OPG secretion from MC3T3-E1 cells (Fig. 5). A limitation of this co-culture system is that the addition of exogenous sRANKL may influence endogenous RANKL expression in osteoblastic cells, which was not directly evaluated in this study. Furthermore, because the two cell types were physically separated by a cell insert membrane, the present experimental design does not allow definitive conclusions regarding the relative contribution of soluble mediators and direct cell–cell interactions. Nevertheless, these findings indicate that *Pg*-OMVs can modulate osteoclast differentiation even under non-contact co-culture conditions. OPG is a key regulator of bone remodeling that inhibits RANKL-mediated osteoclastogenesis. In our co-culture system, *Pg*-OMVs stimulation resulted in a marked reduction in OPG levels in both the upper chamber containing MC3T3-E1 cells and the lower chamber containing RAW264.7 cells. The difference in OPG concentrations between upper and lower chambers suggests that osteoblast-lineage cells may contribute substantially to OPG production under the present conditions; however, differences in cytokine consumption by RAW264.7 cells or diffusion across the membrane cannot be excluded. In parallel, *Pg*-OMVs significantly enhanced the formation of TRAP-positive MNCs and upregulated the expression of essential osteoclast differentiation markers, including NFATc1, DC-STAMP, and Cathepsin K, in RAW264.7 cells. These results suggest that *Pg*-OMVs may influence osteoclast differentiation through modulation of osteoblast–osteoclast communication mediated by soluble factors. *Pg*-LPS was included as a reference control in the co-culture system and induced only a modest decrease in OPG secretion without promoting osteoclast differentiation, indicating a weaker effect compared with *Pg*-OMVs.

We also assessed the role of *Pg*-OMVs in bone resorption in periodontitis using experimental animal models in vivo. The ligature-induced model is widely used to study alveolar bone resorption in periodontitis. In this model, ligature placement induces inflammatory cell infiltration into periodontal tissues. The associations among inflammatory responses, osteoclast formation, and bone resorption are well established in periodontitis. The ligature-induced model shows significant alveolar bone loss due to the stimulation of osteoclast precursor differentiation into osteoclasts^50^. In our previous study, bone loss persisted for 45–90 d^51^. In this study, the OMV administration group exhibited a significantly greater degree of alveolar bone resorption than the healthy control group. Zhang et al.. reported that *Fusobacterium nucleatum*–derived OMVs induce periodontitis-like bone loss independent of ligature placement in a rat model^52^. Consistent with these findings, our results suggest that *Pg*-OMVs alone are sufficient to induce alveolar bone resorption under the present experimental conditions.

Here, the ligature with *Pg*-OMV administration group exhibited a significantly greater degree of alveolar bone resorption than the ligature control group. These findings suggest that *Pg*-OMVs exacerbate bone resorption under periodontitis-like conditions. Fan et al.. and Chen et al.. reported that *Pg*-OMVs promote alveolar bone resorption in vivo^53,54^. Although our results are largely consistent with their findings, direct comparison is difficult due to differences in experimental settings and *Pg*-OMVs concentrations. In their studies, the mechanisms of bone resorption were mainly investigated with a focus on apoptosis, inflammation, and endothelial dysfunction using periodontal ligament cells or vascular endothelial cells. In contrast, the present study examined *Pg*-OMVs–induced bone resorption primarily from the perspective of osteoclast–osteoblast interactions. The RANKL/OPG ratio is known to increase in periodontal tissues under pathological conditions^55^, and a ligature-induced periodontitis model has been shown to significantly upregulate the expression of RANKL, OPG, and pro-inflammatory cytokines such as IL-1β, IL-6, and TNF-α^56^. To date, however, only a limited number of studies have investigated the mechanisms of *Pg*-OMVs–induced alveolar bone resorption with particular emphasis on alterations in the RANKL/OPG axis in osteoblasts and periodontal tissues. In this study, both the ligature with *Pg*-OMV administration and *Pg*-OMV administration groups showed higher RANKL levels and lower OPG levels in periodontal tissues than the healthy control group, indicating an increased RANKL/OPG ratio (Fig. 7). Furthermore, inflammatory cytokine expression, including IL-6 and TNF-α, was elevated in both the ligature with *Pg*-OMV administration and ligature control groups, and partially similar trends were observed in the *Pg*-OMV administration group. Previous studies have also reported that *Pg*-OMVs induce inflammatory cytokines such as IL-6 and TNF-α, which are known to promote osteoclast differentiation^57–61^. Although the relative contributions of ligature and *Pg*-OMVs administration cannot be distinguished in the present experimental design, our findings suggest that *Pg*-OMVs may contribute to inflammatory changes associated with osteoclast differentiation in periodontal tissues.

In line with this interpretation, a previous study reported that *Pg*-OMVs administration increases the proportion of TRAP-positive cells in periodontal tissues^53^. However, histological analyses such as TRAP and H&E staining were not performed in the present study; therefore, changes in osteoclast number, inflammatory cell infiltration, and tissue architecture were not directly evaluated in vivo. Accordingly, the inflammatory and osteoclastic responses should be interpreted in conjunction with µCT findings rather than as direct histological evidence. Importantly, the RANKL/OPG ratio, a critical determinant of osteoclastogenesis, was quantitatively assessed using periodontal tissue extracts, showing an increased ratio particularly in the ligature with *Pg*-OMV administration group. However, because immunohistochemical analyses were not conducted, the cellular sources and spatial localization of RANKL and OPG remain unclear. Similarly, IL-6 and TNF-α were quantitatively measured in periodontal tissue extracts, supporting the involvement of *Pg*-OMVs in inflammatory signaling in vivo. Nevertheless, the absence of immunohistochemical analyses precludes identification of their precise localization and cellular origin. In addition, the in vivo tissue distribution of *Pg*-OMVs was not directly examined, and their precise localization within periodontal tissues remains unclear. ALP activity, a key marker of osteoblast differentiation and mineralization, was not evaluated in alveolar bone tissues in vivo due to tissue processing limitations. Although *Pg*-OMVs suppressed ALP activity in vitro, this limitation restricts interpretation of mineralization at the tissue level. Future studies incorporating histological and immunohistochemical analyses, including H&E and TRAP staining and cell-specific localization of RANKL/OPG and inflammatory cytokines, will be essential to further clarify the mechanisms underlying *Pg*-OMVs–induced periodontal bone resorption.

**Fig. 8:**
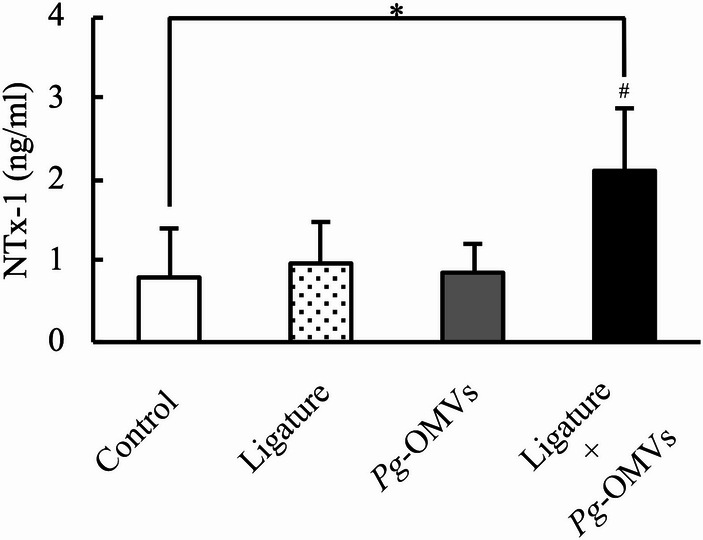
Effect of *Pg*-OMVs on serum NTx-1 levels, a bone resorption marker. Serum NTx-1 concentrations were measured via ELISA. Data are presented as mean ± SD (*n* = 5 per group). *P < 0.05 vs. Control (healthy control group); ^#^*P* < 0.01 vs. Ligature (ligature control group).

The size of *Pg*-OMVs is approximately one to two thousandths that of the bacterial cell body^19^. Owing to their nano-sized structure and membrane encapsulation, OMVs are relatively resistant to enzymatic degradation and have been reported to penetrate tissues that are inaccessible to intact bacteria. Moreover, *Pg*-OMVs can concentrate multiple virulence factors and potentially disseminate from local periodontal sites to systemic circulation. In the present study, serum NTx-1 levels were significantly higher only in the ligature with *Pg*-OMV administration group, indicating that elevation of systemic bone resorption markers was observed under conditions combining ligature-induced periodontal inflammation with *Pg*-OMVs administration (Fig. 8). This finding highlights the potential impact of *Pg*-OMVs on bone resorption under inflammatory conditions.

Several alveolar bone resorption mechanisms have been reported in experimental periodontitis models using *Fn*-OMVs. For example, one study reported that *Fn*-OMVs activate NLR family pyrin domain-containing 3 inflammasomes and impair the mineralization of periodontal ligament stem cells via NF-κB signaling^52^. Another study indicated that *Fn*-OMVs promote macrophage polarization toward the pro-inflammatory M1 phenotype and cytokine release, thereby creating an inflammatory microenvironment in surrounding tissues^40^. However, despite these advances, the mechanisms by which *Pg*-OMVs regulate alveolar bone resorption—particularly through modulation of osteoblast–osteoclast interactions and the RANKL/OPG axis—remain poorly understood. In this context, the present study provides new insight into how *Pg*-OMVs may promote alveolar bone destruction by enhancing osteoclast differentiation and modulating osteoblast function.

In this study, we investigated the effects of *Pg*-OMVs on osteoclastogenesis and alveolar bone resorption in periodontitis by examining changes in the RANKL/OPG balance using murine preosteoblastic and osteoclast precursor cells in vitro, as well as periodontal tissues in an experimental periodontitis model in vivo. However, osteoclastogenesis is also known to be induced via activation of periodontal T and B cells, which serve as important sources of RANKL independently of osteoblasts and other gingival cells^31,32,38^. As the present study primarily focused on osteoblast–osteoclast interactions, the contribution of these immune cell–mediated pathways was not directly evaluated. In addition, OMVs derived from periodontal pathogens have been reported to promote dendritic cell maturation and induce differentiation of naïve CD4⁺ T cells into Th1 and Th17 cells, leading to IL-17 production^62^. Given that IL-17 has been shown to modulate the RANKL/OPG balance and promote osteoclastogenesis in periodontitis^31^, these findings suggest that *Pg*-OMVs may also indirectly influence periodontal bone resorption through cytokine-mediated immune responses. Further studies are required to clarify the effects of *Pg*-OMVs on immune cell–mediated pathways, including IL-17 production and its impact on the RANKL/OPG balance.

In addition, we examined RANK expression in osteoclast precursor cells using both RAW264.7 monoculture and co-culture with MC3T3-E1 cells. We found that *Pg*-OMVs enhanced RANK expression in RAW264.7 cells irrespective of the presence of sRANKL (Fig. S1). These findings suggest that *Pg*-OMVs may modulate the RANK–RANKL–OPG signaling pathway not only by altering RANKL and OPG expression in osteoblastic cells but also by increasing the responsiveness of osteoclast precursors to RANKL through upregulation of RANK.

In this study, we demonstrated both in vitro and in vivo that *Pg*-OMVs are associated with enhanced alveolar bone resorption, accompanied by increased osteoclast differentiation, impaired osteoblast mineralization, and an elevated RANKL/OPG ratio in periodontal tissues. *Pg*-OMVs appear to affect both osteoclasts and osteoblasts through multiple mechanisms in periodontal tissues, thereby potentially disrupting periodontal bone homeostasis and exacerbating alveolar bone loss. Collectively, these findings suggest that *Pg*-OMVs may contribute to the pathogenesis of periodontitis-associated alveolar bone destruction and represent potential therapeutic targets for periodontal disease and related systemic bone disorders.

## Conclusion

In this study, *Pg*-OMVs were associated with enhanced alveolar bone resorption in a ligature-induced periodontitis model, together with an increased RANKL/OPG ratio in periodontal tissues. In vitro, *Pg*-OMVs modulated the RANKL/OPG balance, accompanied by an increase in osteoclast differentiation. These findings suggest that *Pg*-OMVs may contribute to periodontal bone loss. Further studies are warranted to clarify the roles of individual *Pg*-OMVs components in the pathogenesis of periodontitis.

## Methods

### Reagents

Alpha-modified Eagle’s minimal essential medium (α-MEM), ascorbic acid, and β-glycerophosphoric acid were purchased from FUJIFILM Wako Pure Chemical Corporation (Osaka, Japan; catalog numbers 135–15175, 013–12061, and 048–34332, respectively). Fetal bovine serum (FBS) and trypsin-EDTA were purchased from Gibco BRL (Gaithersburg, MD, USA; catalog numbers 12483-012 and 25200-072, respectively), and recombinant murine sRANKL was purchased from PeproTech (Rocky Hill, NJ, USA; catalog number 315 − 11). Mouse monoclonal antibodies against RANKL and OPG were purchased from Novus Biologicals (Centennial, CO, USA; catalog numbers 12A668 and 98A1071, respectively). Rabbit polyclonal antibodies against Cathepsin K, NFATc1, and mouse monoclonal antibody against β-actin were obtained from Abcam (Cambridge, UK; catalog numbers ab19027, ab25916, and ab49900, respectively). Mouse monoclonal antibody against DC-STAMP was obtained from EMD Millipore (Temecula, CA, USA; catalog number MABF39-I), and against RANK was obtained from Santa Cruz Biotechnology Inc., (Santa Cruz, CA, USA, catalog number sc-390655). Horseradish peroxidase (HRP)-conjugated goat anti-rabbit IgG, goat anti-rat IgG, and horse anti-mouse IgG antibodies were purchased from Cell Signaling Technology (Beverly, MA, USA; catalog numbers 7074, 7077, and 7076, respectively). LPS from *Pg* (*Pg*-LPS) was purchased from InvivoGen (San Diego, CA, USA).

### OMV isolation and endotoxin activity assay

*Pg* ATCC 33,277 (type strain) was anaerobically cultured in the brain heart infusion broth (Becton Dickinson, Sparks, MD, USA) supplemented with 5 µg/mL hemin and 1 µg/mL menadione for two days. *Pg*-OMVs were isolated using a previously established protocol^29,63^.

Endotoxin activity of OMVs was determined via colorimetric LAL assay (Limulus Color KY Test; FUJIFILM Wako Pure Chemical Corporation), according to the manufacturer’s instructions. In this study, the concentration of *Pg*-OMVs added to the cells was set based on the concentration of *Pg*-LPSs showing an equal level of endotoxin activity.

### Cell culture

RAW264.7 murine macrophage cell line was obtained from the American Type Culture Collection (Rockville, MD, USA), and MC3T3-E1 murine preosteoblastic cell line was obtained from Riken Cell Bank (Tsukuba, Japan). The cells were seeded in 6- or 12-well plates (IWAKI, Chiba, Japan) at a density of 10,000 cells/cm² and cultured in α-MEM supplemented with 10% FBS at 37 °C in a humidified atmosphere of 5% CO₂ and 95% air. RAW264.7 cells were differentiated into osteoclasts using sRANKL (50 ng/mL) for 5 days, as described by Zhang et al.^64^. MC3T3-E1 cells were cultured in a calcification-inducing medium (α-MEM supplemented with 10% FBS, 50 µg/mL ascorbic acid, and 2 mmol/L β-glycerophosphate) for 24 days to induce osteoblastic differentiation and mineralization^65^. The culture medium was changed every 72 h. Specific treatment conditions, including *Pg*-OMVs and *Pg*-LPS exposure, are described in each corresponding experimental section.

### Cell viability assay

Cell viability was examined using Cell Counting Kit-8 (catalog no. CK04; Dojindo, Kumamoto, Japan) according to the manufacturer’s instructions. Briefly, RAW264.7 and MC3T3-E1 cells were seeded at a density of 5,000 cells/well in 96-well plates (SUMITOMO BAKELITE, Tokyo, Japan) and cultured in growth medium (α-MEM supplemented with 10% FBS). After 24 h of incubation to allow cell attachment, RAW264.7 cells were stimulated with sRANKL (50 ng/mL). Both RAW264.7 and MC3T3-E1 cells were treated with medium alone or *Pg*-OMVs (5–200 ng/mL) for 48 h, with exposure defined by concentration (ng/mL) in a fixed culture volume to ensure consistency across plate formats. Subsequently, Cell Counting Kit-8 solution was added, and the cells were incubated for 2 h. Absorbance was measured at 450 nm using a microplate reader (iMark; Bio-Rad, Hercules, CA, USA). Cell viability was expressed as a percentage relative to the medium-alone control. All experiments were performed with three technical replicates per condition and repeated in three independent experiments. For statistical analysis, the mean of the technical replicates was treated as a single data point (*n* = 3).

### TRAP staining

To determine the effect of *Pg*-OMVs on osteoclast differentiation, RAW264.7 cells were cultured in the presence or absence of sRANKL (50 ng/mL) and treated with *Pg*-OMVs (0, 10, and 100 ng/mL) for 5 days. Osteoclast formation was then evaluated by TRAP staining using a TRAP/ALP stain kit (catalog number 294–67001; FUJIFILM Wako Pure Chemical Corporation) according to the manufacturer’s instructions. TRAP-positive MNCs with ≥ 3 nuclei were counted by an independent examiner in a blinded manner using a phase-contrast microscope at 100× magnification. Statistical analysis was performed using the sRANKL-unstimulated control (0 ng/mL sRANKL, 0 ng/mL *Pg*-OMVs) and the sRANKL-stimulated control (50 ng/mL sRANKL, 0 ng/mL *Pg*-OMVs) as reference groups, as indicated in the figure legends. This procedure was repeated three times using triplicate RAW264.7 cell cultures.

### Western blotting

Cells lysates were lysed using 20 or 50 µL of radioimmunoprecipitation assay (RIPA) lysis buffer (catalog number sc-24948; Santa Cruz Biotechnology Inc). The cell lysates were subjected to Western blotting, as previously described^66^. Briefly, equal amounts of proteins (30 µg) were denatured and separated via 10% sodium dodecyl sulfate polyacrylamide gel electrophoresis and transferred to polyvinylidene difluoride membranes (catalog numbers 4561096 and 1704156; Bio-Rad). The membranes were either cut horizontally into sections or subjected to antibody stripping and reprobing. After blocking with 5% non-fat dry milk in 50 mM Tris-buffered saline (pH 7.6) containing 0.05% Tween 20, the membranes were incubated overnight at 4 °C with diluted primary antibodies against RANKL, OPG, Cathepsin K, DC-STAMP, NFATc1, and RANK (all at 1:1000 dilution), followed by incubation with HRP-conjugated secondary antibodies (1:2000 dilution) at room temperature for 1 h. Anti-β-actin antibody (1:5000 dilution) was used as a loading control. After washing, the blots were developed using the Amersham ECL Western Blotting Detection Reagents and visualized using Image Quant LAS 500 (GE Healthcare, Little Ckalfont, UK). Densitometric analysis was performed using ImageJ software, and band intensities were normalized to β-actin. For in vitro experiments, the entire procedure was independently repeated three times (*n* = 3).

### Quantitative reverse transcription-polymerase chain reaction (qRT-PCR)

To investigate the effects of *Pg*-OMVs on RANKL and OPG mRNA expression, MC3T3-E1 cells were cultured with or without *Pg*-OMVs (10–150 ng/mL) in a calcification-inducing medium for 48 h. Total RNA was isolated from the cells using NucleoSpin RNA (TaKaRa Bio, Otsu, Japan), and cDNA was synthesized from 500 ng of total RNA using the PrimeScript RT Master Mix (Perfect Real Time; TaKaRa Bio). qRT-PCR was performed using the CFX96 Real-Time PCR Detection System (Bio-Rad). Template cDNA was mixed with the SYBR Green Supermix (catalog number: 1725270; Bio-Rad), distilled water, and primers. The reaction conditions were as follows: 95 °C for 30 s, followed by 40 cycles of 95 °C for 10 s and 60 °C for 30 s. The following primer sets were used: *RANKL-F*, 5ʹ-TGGAAGGCTCATGGTTGGAT-3ʹ; *RANKL-R*, 5ʹ-CATTGATGGTGAGGTGTGCAA-3ʹ; *OPG-F*, 5ʹ-CAGAGAAGCCACGCAAAAGTG-3ʹ; *OPG-R*, 5ʹ-AGCTGTGTCTCCGTTTTATCCT-3ʹ; glyceraldehyde-3-phosphate dehydrogenase (*GAPDH*)-*F*, 5ʹ-AACTTTGGCATTGTGGAAGG-3ʹ; *GAPDH-R*, 5ʹ-ACACATTGGGGGTAGGAACA-3ʹ. Relative mRNA levels of target genes were normalized to those of *GAPDH* as an internal control.

### Evaluation of ALP activity and calcified nodule formation in culture

MC3T3-E1 cells were cultured with or without *Pg*-OMVs (10–150 ng/mL) in a calcification-inducing medium for 24 d. After the culture period, the cells were collected and lysed, and ALP activity was measured using a fluorescence detection kit (LabAssay ALP; catalog number 633–51021; Shibukawa, Gunma, Japan) during the culture period. Furthermore, to observe ALP activity in the culture plates, the cells were washed with phosphate-buffered saline (PBS), fixed with 10% neutral-buffered formalin, and stained using the TRAP/ALP stain kit (FUJIFILM Wako Pure Chemical Corporation). The stained areas were observed using the National Institutes of Health (NIH) image software version 1.62 (Bethesda, MD), as previously described^67^. To assess calcified nodule formation in the plates, the cells were washed, fixed in the same manner, and stained using the von Kossa technique^68,69^. The area of calcified nodules, stained as dark dots, was determined using the NIH image software.

### Co-culture system

RAW264.7 cells and MC3T3-E1 cells were co-cultured using a cell insert system. RAW264.7 cells were seeded at a density of 10,000 cells/cm² onto the bottom of 24-well plates (lower chamber; Falcon, New York, USA) and cultured in α-MEM supplemented with 10% FBS. After 24 h, MC3T3-E1 cells were seeded at the same density onto cell culture inserts with 0.4 μm pore size filters (upper chamber; Falcon). This insert-based system allowed the two cell types to be co-cultured without direct cell–cell contact, enabling paracrine interactions. At the start of co-culture, sRANKL (50 ng/mL) was added to induce osteoclast differentiation of RAW264.7 cells, together with *Pg*-OMVs (0, 10, and 100 ng/mL) or *Pg*-LPS (100 ng/mL). As endogenous sRANKL released from MC3T3-E1 cells was below the detection limit under our experimental conditions, exogenous sRANKL was added to ensure stable osteoclastogenic conditions and enable quantitative evaluation of RANKL/OPG regulation. The culture medium was replaced every 2 days, and stimulation with sRANKL and *Pg*-OMVs or *Pg*-LPS was repeated. The co-culture was maintained for a total of 5 days.

After 5 days of co-culture, culture supernatants were collected from both the upper and lower chambers. The concentration of OPG in the culture supernatants was quantified using a sandwich enzyme-linked immunosorbent assay (ELISA) kit (Catalog number DY459; R&D Systems, Minneapolis, MN, USA) according to the manufacturer’s instructions. Furthermore, osteoclast formation was evaluated by TRAP staining, and TRAP-positive MNCs containing three or more nuclei were counted as osteoclasts. In addition, Western blot analysis was performed to assess the protein expression levels of NFATc1, DC-STAMP, Cathepsin K, and RANK in RAW264.7 cells.

### Animals

All animal experiments were approved by the Animal Research Control Committee of the Tokushima University Graduate School (T2021-35) and were performed in accordance with the relevant guidelines and regulations, as well as the Animal Research: Reporting of In Vivo Experiments (ARRIVE) guidelines of the National Centre for the Replacement, Refinement, and Reduction of Animals in Research. Twenty male seven-week-old Wistar rats (220–230 g; Charles River Laboratories Japan, Inc., Yokohama, Japan) were housed in a temperature- and humidity-controlled room (23 ± 1 °C and 60 ± 5% relative humidity) under a 12/12-h light/dark cycle and provided standard rodent chow and water ad libitum.

### Alveolar bone resorption experiments

Twenty rats were randomly divided into four groups (*n* = 5/group) as follows: (1) healthy control group (rats were injected with 10 µL of PBS into the subperiosteum at the buccal gingiva of the maxillary right second molar every other day for 16 d), (2) OMV administration group (rats were injected with 10 µL of *Pg*-OMVs [1.5 µg/µL] into the subperiosteum at the buccal gingiva of the maxillary right second molar every other day for 16 d), (3) ligature control group (experimental periodontitis was induced using ligatures, and rats were injected with the same volume of PBS into the subperiosteum at the buccal gingiva of the maxillary right second molar every other day for 16 d), and (4) ligature with OMV administration group (experimental periodontitis was induced using ligatures, and rats were injected with 10 µL of *Pg*-OMVs [1.5 µg/µL] into the subperiosteum at the buccal gingiva of the maxillary right second molar every other day for 16 d). Experimental periodontitis was induced in rats as previously described^51,56^. Briefly, cervical area of the right second molar of the rat maxilla was ligated using a nylon thread (No. 5 − 0; Natsume Corporation, Tokyo, Japan) under anesthesia with sodium pentobarbital (30 mg/kg). The ligature was knotted on the buccomesial side to ensure that it remained intact during the experimental period and checked every other day to confirm subgingival placement. The left second molar of the rat maxilla was used as the non-ligatured control (untreated). The number of rats was set via size calculation based on a previous report^70^.

### Micro-computed tomography (µCT) analysis of the alveolar bone

On day 16, all rats were euthanized with an overdose of sodium pentobarbital (120 mg/kg, intraperitoneal injection), followed by cervical dislocation to ensure death. The maxillae and peripheral blood were then collected. The alveolar bone specimens were immediately fixed with 10% neutral-buffered formalin, and alveolar bone resorption degree was analyzed using a µCT system (SkyScan 1176; Bruker, Billerica, MA, USA), as previously described^51,71–74^. The distance from buccal CEJ to ABC of the second molar was measured in the frontal section as a marker of bone height. To ensure reproducibility of the alignment of the µCT image, the buccal cusp tip of the second molar was placed such that they superimposed on the corresponding palatal cusp tip. The distance between CEJ and ABC was measured at four points on the buccal side of the second molar, including the mesial, distal, and middle (furcation) sites, and the average was calculated. Furthermore, bone morphometric parameters, including bone volume fraction (BV/TV), were calculated from the furcation area on the buccal side of the second molar using CTan software (Bruker) to evaluate periodontal bone tissue in each group.

### Analysis of gingival biopsy from the rat maxilla

After harvesting the maxillae, gingival biopsy samples surrounding the second molar were quickly excised and frozen in liquid nitrogen. Tissue lysates were isolated from the gingival tissues as previously described^75^, with slight modifications. Briefly, gingival tissues were homogenized using BioMasher II (catalog number 380–21591; FUJIFILM Wako Pure Chemical Corporation), a homogenizer, and lysed using a 100 µL of RIPA lysis buffer. Then, the lysates were subjected to Western blotting using primary antibodies against RANKL and OPG, and production levels of IL-6 and TNF-α were determined using the respective ELISA kits (catalog numbers DY506-05 and DY510-05, respectively; R&D Systems, Inc.), according to the manufacturer’s instructions. Protein samples were obtained from five animals per group (*n* = 5), and each sample was analyzed individually by Western blot.

Sera were prepared from the blood samples, and NTx-1 (cross-linked N-telopeptide of collagen type I) levels were determined using an enzyme-linked immunosorbent assay kit (catalog number LS-F21857; Life Span BioSciences, Inc., Seattle, WA, USA), according to the manufacturer’s instructions.

### Statistical analyses

Statistical analyses were conducted using SPSS Statistics version 20 (Armonk, NY, USA). Data are presented as mean ± standard deviation (SD). Normality was assessed using the Anderson–Darling test. For multiple group comparisons, one-way analysis of variance (ANOVA) followed by the Tukey–Kramer post hoc test was applied. Densitometric data from Western blot analyses, obtained from independent biological replicates, and other quantitative data were included in these analyses. A *P* value < 0.05 was considered statistically significant.

## Supplementary Information

Below is the link to the electronic supplementary material.

Supplementary Material 1

Supplementary Material 2

Supplementary Material 3

Supplementary Material 4

Supplementary Material 5

Supplementary Material 6

Supplementary Material 7

## Data Availability

Data generated/analyzed in this study are available upon reasonable request from the corresponding author.

## Declarations

### Use of large language models (LLMs)

A large language model (ChatGPT, OpenAI) was used for minor English language editing during the revision of this manuscript.
