# Supplementary material for: Porphyromonas gingivalis outer membrane vesicles promote alveolar bone resorption by increasing the local RANKL/OPG ratio in experimental periodontitis model rats
Source: Sci Rep. 2026 Apr 5;16:17570. doi: 10.1038/s41598-026-46625-4 (PMC13243619; doi:10.1038/s41598-026-46625-4)
Supplement: Supplementary file 7 — Supplementary Material 7 [file 41598_2026_46625_MOESM7_ESM.docx]

**Fig. S1.** Effects of *Pg*-OMVs on RANK expression in RAW264.7 cells. (**A, B**) RANK expression in RAW264.7 cells cultured under the same conditions as in Fig. 2C–E was analyzed by Western blotting. β-actin served as a loading control. All bands were derived from the same gel; membranes were either cut horizontally after transfer or stripped and reprobed with the indicated antibodies. Data are presented as the mean ± SD from three independent biological replicates (n = 3). **P* < 0.05 vs. the sRANKL-unstimulated control (0 ng/mL sRANKL, 0 ng/mL *Pg*-OMVs); #*P* < 0.05 vs. the sRANKL-stimulated control (50 ng/mL sRANKL, 0 ng/mL *Pg*-OMVs). (**C**) RANK expression in the RAW264.7–MC3T3-E1 co-culture system was analyzed by Western blotting. RAW264.7 cells in the lower chamber were cultured under the same conditions as in Fig. 5. A representative image is shown, and similar results were obtained in two independent experiments. β-actin served as a loading control. All bands were derived from the same gel; membranes were either cut horizontally after transfer or stripped and reprobed with the indicated antibodies.

**Fig. S2.** Full-length blots corresponding to Fig. 2C. Full-length blots are presented. Cropped regions used in the figures are indicated by red boxes. The membrane was cut at approximately 75 kDa, and the cutting position is indicated by a dashed line. The upper portion was probed with the antibody against NFATc1, while the lower portion was sequentially probed with antibodies against Cathepsin K, DC-STAMP, and β-actin in that order. Between each detection on the lower portion, the membrane was stripped before reprobing with the next antibody.

**Fig. S3.** Full-length blots corresponding to Fig. 3G. Full-length blots are presented. Cropped regions used in the figures are indicated by red boxes. The membrane was sequentially probed with antibodies in the following order: sRANKL, OPG and β-actin. After each detection, the membrane was stripped before reprobing with the next antibody to confirm equal loading. Uniform adjustments of brightness and contrast were applied across the entire images.

**Fig. S4.** Full-length blots corresponding to Fig. 5E. Full-length blots are presented. Cropped regions used in the figures are indicated by red boxes. The membrane was cut at approximately 75 kDa, and the cutting position is indicated by a dashed line. The upper portion was probed with the antibody against NFATc-1, while the lower portion was sequentially probed with antibodies against Cathepsin K, DC-STAMP, and β-actin in that order. Between each detection on the lower portion, the membrane was stripped before reprobing with the next antibody.

**Fig. S5.** Full-length blots corresponding to Fig. 7A. Full-length blots are presented. Cropped regions used in the figures are indicated by red boxes. The membrane was sequentially probed with antibodies in the following order: sRANKL, OPG, and β-actin. After each detection, the membrane was stripped before reprobing with the next antibody to confirm equal loading. Uniform adjustments of brightness and contrast were applied across the entire images.

**Fig. S6.** Full-length blots corresponding to Fig. S1. (**A**) Full-length blots for Fig. S1A, derived from the same membrane used for Fig. 2C. (**B**) Full-length blots for Fig. S1C, derived from the same membrane used for Fig. 5E. Cropped regions used in the corresponding figures are indicated by red boxes. Brightness and contrast were adjusted uniformly across the entire images. Uniform adjustments of brightness and contrast were applied across the entire images.
